# Exploring Verbal Fluency Strategies among Individuals with Normal Cognition, Amnestic and Non-Amnestic Mild Cognitive Impairment, and Alzheimer’s Disease

**DOI:** 10.3390/medicina59101860

**Published:** 2023-10-19

**Authors:** Styliani Bairami, Vasiliki Folia, Ioannis Liampas, Eva Ntanasi, Panayiotis Patrikelis, Vasileios Siokas, Mary Yannakoulia, Paraskevi Sakka, Georgios Hadjigeorgiou, Nikolaos Scarmeas, Efthimios Dardiotis, Mary H. Kosmidis

**Affiliations:** 1 Lab of Cognitive Neuroscience, School of Psychology, Aristotle University of Thessaloniki, University Campus, 54124 Thessaloniki, Greece; 2 Department of Neurology, University Hospital of Larissa, School of Medicine, University of Thessaly, Mezourlo Hill, 41110 Larissa, Greece; 3 Department of Nutrition and Dietetics, Harokopio University, 70 El. Venizelou, 17671 Athens, Greece; 4 1st Department of Neurology, Aiginition Hospital, National and Kapodistrian University of Athens Medical School, Vassilissis Sofias Ave 72, 11528 Athens, Greece; 5 Athens Alzheimer’s Association, 89 M. Mousourou & 33 Stilponos St, 11636 Athens, Greece; 6 School of Medicine, University of Cyprus, 93 Agiou Nikolaou St, Engomi, Nicosia 2408, Cyprus; 7 Taub Institute for Research in Alzheimer’s Disease and the Aging Brain, The Gertrude H. Sergievsky Center, Department of Neurology, Columbia University, 710 West 168th St, New York, NY 10032, USA

**Keywords:** verbal fluency strategies, clustering, switching, intrusions, perseverations, continuum of neurocognitive decline, mild cognitive impairment, amnestic MCI, non-amnestic MCI

## Abstract

*Background and Objectives:* The present study explored the utilization of verbal fluency (VF) cognitive strategies, including clustering, switching, intrusions, and perseverations, within both semantic (SVF) and phonemic (PVF) conditions, across a continuum of neurocognitive decline, spanning from normal cognitive ageing (NC) to mild cognitive impairment (MCI) and its subtypes, amnestic (aMCI) and non-amnestic (naMCI), as well as AD. *Materials and Methods:* The study sample was derived from the Hellenic Longitudinal Investigation of Aging and Diet (HELIAD) cohort. The sample included 1607 NC individuals, 146 with aMCI (46 single-domain and 100 multi-domain), 92 with naMCI (41 single-domain and 51 multi-domain), and 79 with AD. Statistical analyses, adjusting for sex, age, and education, employed multivariate general linear models to probe differences among these groups. *Results:* Results showed that AD patients exhibited poorer performance in switching in both VF tasks and SVF clustering compared to NC. Similarly, the aMCI group performed worse than the NC in switching and clustering in both tasks, with aMCI performing similarly to AD, except for SVF switching. In contrast, the naMCI subgroup performed similarly to those with NC across most strategies, surpassing AD patients. Notably, the aMCI subgroup’s poor performance in SVF switching was mainly due to the subpar performance of the multi-domain aMCI subgroup. This subgroup was outperformed in switching in both VF tasks by the single-domain naMCI, who also performed better than the multi-domain naMCI in SVF switching. No significant differences emerged in terms of perseverations and intrusions. *Conclusions:* Overall, these findings suggest a continuum of declining switching ability in the SVF task, with NC surpassing both aMCI and AD, and aMCI outperforming those with AD. The challenges in SVF switching suggest executive function impairment associated with multi-domain MCI, particularly driven by the multi-domain aMCI.

## 1. Introduction

The verbal fluency (VF) task has been extensively studied as a potential screening tool for differentiating individuals within the context of Alzheimer’s disease (AD) progression, encompassing the continuum from normal cognitive ageing (NC) to mild cognitive impairment (MCI), as well as AD itself [1,2,3,4,5]. The majority of research primarily focuses on the total number of words produced within a predetermined time frame as the typical evaluation method [6,7] in the two task conditions: the semantic verbal condition (SVF), in which participants generate as many words as possible within a specific category; and the phonemic verbal fluency (PVF) condition, in which participants produce as many words as possible starting with a specific letter. Undoubtedly, the use of total word count scoring, particularly within the SVF condition, has proven a valuable tool for distinguishing between older healthy individuals and those developing MCI or dementia [1,4,8,9]. Recently, research has expanded its scope to investigate the total word count scoring in MCI subtypes, specifically amnestic (aMCI) and non-amnestic (naMCI) single subtypes (aMCI-SD, naMCI-SD) as well as multi-domain subtypes of MCI (aMCI-MD and naMCI-MD). However, results have been inconsistent. Some studies have reported similar performance between both groups on VF tasks [10], while others have suggested that naMCI individuals perform better than aMCI individuals in SVF, but not in the PVF condition [11,12]. There are also studies in which naMCI individuals exhibited poorer performance compared to those with aMCI in both SVF and PVF conditions [13,14]. These variations could, in part, be attributed by the proportion of participants with single- and multi-domain MCI in the samples, as previous research has postulated that multi-domain participants tend to exhibit poorer performance than their single-domain counterparts in language-related tasks, including verbal fluency [4].

In addition to total word production, valuable insights can also emerge from the cognitive strategies employed in VF, namely clustering, switching techniques, and errors, particularly the frequency of intrusions and perseverations. The utilization of VF strategies has the potential to elucidate the specific cognitive functions that may be impaired and that contribute to a performance level below established norms [13,15,16,17,18]. Clustering involves generating words within semantic or phonemic subcategories tapping into the realm of semantic memory knowledge—a temporal-lobe-based process. In contrast, switching refers to the proficiency in transitioning between clusters efficiently, and is thus more closely linked to executive functions (e.g., updating, flexibility), which are orchestrated within the frontal lobe [15]. Intrusions encompass words unrelated to the specified category, whereas perseverations involve repeated words. Perseverations seem to emerge from deficits of the central executive component of the working memory and a failure of the ability to inhibit [19,20], both of which are thought to rely on the frontal lobes [21,22]. Intrusions, on the other hand, are hypothesized to rely on semantic retrieval [23].

In contrast to the research based on total word count, the potential of the cognitive strategies used in VF tasks in differentiating individuals across a continuum of neurocognitive decline and in particular across the MCI subtypes remains unclear. Previous research has suggested that switching in SVF can distinguish AD patients from cognitively normal individuals [17,24], but it may not effectively differentiate aMCI patients (both single and multi-domain) from NC or AD individuals [25]. Also, both Price et al. [18] and Murphy et al. [8] demonstrated no disparity in the frequency of switches between aMCI when compared to control groups. This suggests that individuals with MCI might not yet present with substantial executive dysfunction. With respect to clustering, individuals with amnestic MCI (aMCI) produced smaller [8,18] or fewer clusters compared to controls, suggesting early semantic memory impairment associated with AD [26]. When MCI subtypes were investigated, however, individuals with multi-domain aMCI and dementia displayed reduced clustering and switching ability [27,28], indicating a progressive decline in semantic memory and executive functions. There are limited studies involving non-amnestic MCI or those that compare patients with single- and multi-domain MCI [11,12,13,14]. Only one of these studies has explored VF strategies in these groups [27] and found that the single-domain aMCI group performed similarly to those with normal cognition on all VF measures, while multi-domain aMCI produced reduced total words and switching in both VF tasks. The naMCI had the same pattern, but only on PVF. Employing the VF strategy components to detect differences among the various MCI subtypes and individuals across the spectrum from normal cognition to Alzheimer’s disease can significantly contribute to the diagnostic process by bolstering its specificity and predictive capability [24,29,30,31,32]. Additionally, the utilization of MCI subtypes can play a crucial role in distinguishing subcategories that demonstrate heightened clinical homogeneity, thereby enhancing both diagnostic utility and validity, as pointed out by Kendell et al. [33]. However, despite its potential importance in comprehending the cognitive decline pattern during healthy aging, MCI, and the AD, the literature concerning this field of verbal fluency tasks remains scarce.

Given the limited exploration of VF strategies beyond the typical evaluation method for VF, and in view of the inconsistencies in the existing literature, the objective of the current study was to evaluate the utility of the VF strategies used, namely switching, clustering, intrusions, and perseverations, in distinguishing among individuals within the complete AD spectrum, ranging from normal cognitive ageing (NC) individuals, MCI individuals, its various subtypes (aMCI-SD, naMCI-SD, aMCI-MD, and naMCI-MD), and AD patients. This investigation was conducted using a sizable and representative sample of older Greek individuals derived from the HELIAD (Hellenic Longitudinal Investigation of Aging and Diet) cohort [34]. All analyses accounted for measures of global cognition and important sociodemographic parameters that may confound language impairment and dementia-MCI development.

## 2. Materials and Methods

Our aim in the present study was to investigate verbal fluency strategies among older individuals with varying cognitive levels. Specifically, our focus was on individuals with normal cognition (NC), amnestic mild cognitive impairment (aMCI), non-amnestic mild cognitive impairment (naMCI), and Alzheimer’s disease (AD). We also sought to quantify pairwise differences. The study adhered to the STROBE (Strengthening the Reporting of Observational Studies in Epidemiology) recommendations [35] and utilized data from the HELIAD (Hellenic Longitudinal Investigation of Aging and Diet) cohort, a population-based study designed to explore neuropsychiatric disorders in the aging Greek population [32,36,37]. Ethical approval was obtained from the Institutional Ethics Review Boards of the Kapodistrian University of Athens and the University of Thessaly, and all participants or their surrogates provided informed consent.

Participants were randomly selected from the senior (>64 years) registries of two Greek municipalities, Marousi (Athen’s area) and Larissa (province of Thessaly). Comprehensive baseline and follow-up assessments, each lasting approximately 2.5 h, were conducted by a team of expert neurologists, neuropsychologists, and dieticians. These assessments took place in various settings, including participants’ residences, day care centers for the elderly, municipal public health clinics, among others. Data were collected directly from the participants or, when necessary, from their caregivers, such as first-degree relatives. The present study utilized the baseline evaluations carried out as part of the HELIAD study, which were conducted from 2009 to 2015.

### 2.1. Study Assessments

The study assessments involved a range of evaluations conducted by certified neurologists, trained neuropsychologists, and dietitians. These assessments included structured interviews, physical examinations, laboratory investigations, neuropsychological assessments, and the completion of validated questionnaires.

For the purposes of this investigation, we focused on verbal fluency tasks. These tasks included a category fluency task (SVF) and a letter fluency task (PVF) [32,38]. Participants were instructed to generate as many distinct words as possible, either following the announcement of a semantic category (objects) or starting with a specific Greek letter ([a], alpha). No specific word search and production instructions were provided to ensure participants employed various cognitive strategies. However, they were instructed not to include proper nouns or repetitions. Semantic and phonemic clusters, as well as transitions, were computed according to established guidelines [38,39]. In summary, regarding SVF, semantic clusters were identified as sets of three or more consecutive words belonging to the same semantic subcategory or as pairs of consecutive words exhibiting a strong association in the Greek language. Additionally, semantic switches were calculated by subtracting the total number of words belonging to a semantic cluster from the total word production count and then adding the number of semantic clusters to that result. Regarding PVF, phonemic clusters were defined as three or more consecutive words beginning with the same two letters, sharing the same sound, or two consecutive words that were considered strong pairs as they are commonly associated with each other. Phonemic switches were calculated by subtracting the sum of words belonging to a phonemic cluster from the total phonemic word production. This result was then added to the number of phonemic clusters [38,39]. Successive words stemming from the same root (such as act–action–acting) were regarded as repetitions. Lastly, words that were either identical to, or variations of, a word previously provided (i.e., compound words) were treated as perseverations.

Diagnostic classifications for participants’ cognitive status were determined through expert consensus meetings involving senior neurologists (E.D., G.M.H., and N.S.) and neuropsychologists (M.H.K.). A comprehensive explanation of the approach utilized can be found in other sources [37,40]. In brief, dementia and AD diagnoses followed the criteria outlined in the Diagnostic and Statistical Manual of Mental Disorders -IV-text revision [41] and the National Institute of Neurological and Communicative Disorders and Stroke/Alzheimer Disease and Related Disorders Association criteria [42], respectively. The diagnosis of MCI and its subtypes followed the Petersen criteria [43], with amnestic MCI indicating isolated amnestic impairment or multi-domain impairment involving memory function and non-amnestic MCI encompassing language, attention, executive, or visuo-perceptual impairment or any combination thereof (excluding memory impairment).

### 2.2. Outcome Measures and Statistical Analysis

Differences in the VF strategies among (and between) individuals based on cognitive status (NC, aMCI, naMCI, AD) were investigated using adjusted multivariate general linear models (GLMs). The primary multivariate GLM included the strategies used in SVF and PVF as dependent variables, adjusted for sex (treated as a categorical variable) as well as age in years and years of formal education (treated as scale variables). Post hoc between-group comparisons were performed according to the Bonferroni correction. An exploratory adjusted multivariate GLM was also conducted in order to investigate differences in VF strategies among single and multi-domain amnestic and naMCI. VF cognitive strategies were analyzed using an interchangeable statistical approach as described above (the Bonferroni correction was once again implemented). All statistical analyses were performed using the IBM SPSS Statistics Software Version 25 (Chicago, IL, USA). The conventional 5% threshold of significance was implemented.

## 3. Results

### 3.1. Baseline Characteristics and Missing Data

The HELIAD battery included 1984 participants at baseline. Out of these, 36 individuals were excluded due to missing data, resulting in inconclusive diagnosis. Additionally, 24 participants were excluded owing to the identification of other dementias (9 individuals were diagnosed with vascular dementia, 8 with Parkinson’s dementia or dementia with Lewy Bodies, and 7 with less common entities). The remaining 1924 participants were classified as follows: 1607 with NC, 146 with aMCI, 92 with naMCI, and 79 with AD. Table 1 presents descriptive data for baseline demographic information and verbal fluency measurements per group. The mean age of our sample was 73.82 years (±SD) ±5.43, ranging between 65.03 and 99.65 years. The majority of the participants were female (59.5%). The mean years of education of our sample was 7.98 years (±SD) ±4.93, ranging between 0 and 21 years of education. Among MCI participants, there were 46 individuals with isolated aMCI, 100 with multi-domain aMCI, 41 with isolated naMCI, and 51 with multi-domain naMCI. Extensive details with respect to the baseline characteristics of these participants are provided elsewhere [40].

### 3.2. Differences in Verbal Fluency Strategies among Groups Based on Cognitive Status

**Figure 1:**
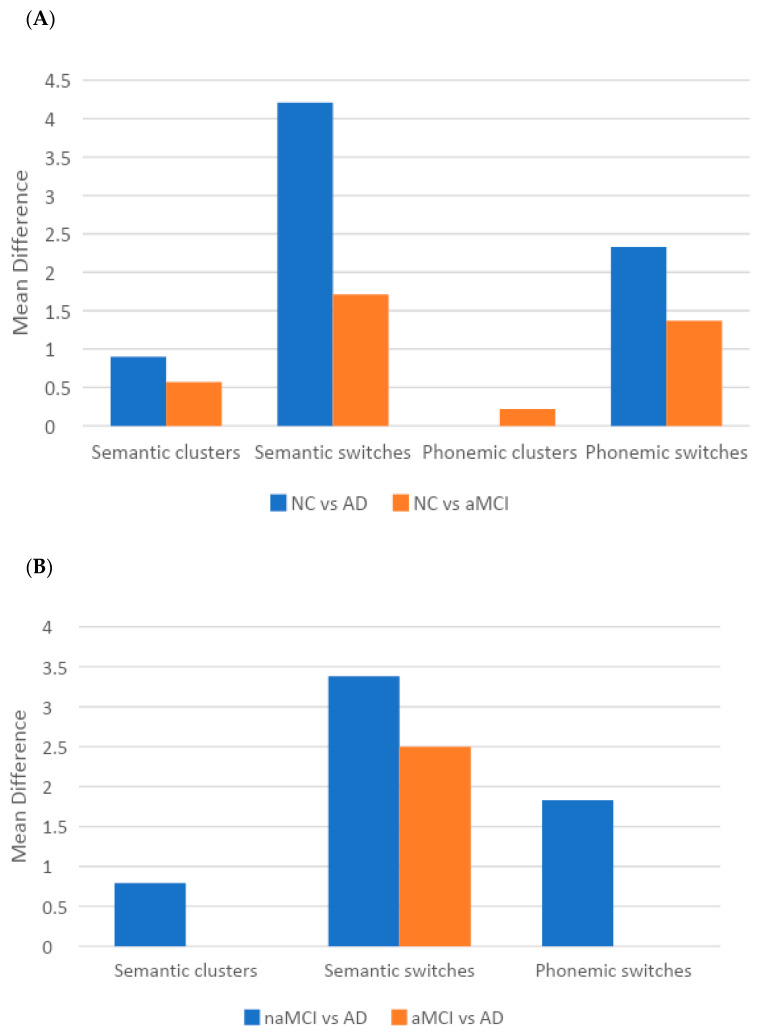
Schematic illustration of the significant adjusted differences (**A**) between NC and AD; NC and aMCI individuals and (**B**) between naMCI and AD; aMCI and AD, in the main eight verbal fluency strategies. Differences conventionally represent impaired performance of the second relative to the first group of participants as quoted in clarification of the different shadings at the bottom of each figure. A total of 1814 participants with available data for all dependent and independent variables (1519 NC, 138 aMCI, 90 naMCI, 67 AD) underwent analysis using multivariate general linear models (GLMs), adjusted for sex, age, and education. NC: normal cognition; aMCI: amnestic mild cognitive impairment; naMCI: non-amnestic MCI; AD: Alzheimer’s disease.

Table 2 contains a comprehensive depiction of the differences in the VF strategies among (and between) groups based on cognitive status, adjusted for the mean age and education of our sample as well as the average effect of sex. Specifically, individuals with aMCI produced fewer clusters and switches in both tasks when compared to those with NC. Similarly, AD patients were surpassed by those with NC in SVF clusters and switches, as well as PVF switches. However, when naMCI participants were compared to those with NC, no significant differences were found. Notably, the differences between individuals with NC and AD were more prominent than those observed between NC and MCI groups (Figure 1a).

No differences were observed between the two MCI groups in terms of VF strategies either. There was only a trend in SVF clusters in favor of the naMCI, relative to the aMCI group. Regarding aMCI and AD, participants in the former group outperformed the latter only in the number of switches in the SVF task. Interestingly, the naMCI advantage over that of the AD group indicated a pattern similar to that of NC and AD: those with naMCI produced more clusters in SVF and more switches in both tasks compared to AD patients. The advantage of naMCI over the AD group in SVF switches was more pronounced than that of aMCI over AD (Figure 1b). No statistically significant differences were detected among any of the groups with respect to errors and perseverations.

### 3.3. Differences in Verbal Fluency Strategies among MCI Subtype Groups

The differences in VF strategies among single and multi-domain amnestic and naMCI groups, as displayed in Table 3, indicate the superiority of single-domain participants’ performances in VF switches. In specific, individuals with single-domain naMCI surpassed those with multi-domain aMCI in both PVF and SVF switches. No differences emerged when participants with single- and multi-domain aMCI were compared. Additionally, individuals with single-domain naMCI performed better than those with multi-domain naMCI in SVF switches. No statistically significant differences were observed among the groups with respect to errors and perseverations. These differences were adjusted for the mean age and education of our sample, as well as the average effect of sex.

## 4. Discussion

In the present study, we investigated differences in VF strategies among NC elderly individuals and MCI and AD patients focusing on the discrepancies across the different MCI subtypes: amnestic and naMCI, as well as single- and multi-domain MCI. Overall, the data presented here reaffirm the utility of those strategies as a useful screening tool for distinguishing between normal cognition, MCI, and AD, as previous research has proposed [17,24,25]. These differences are particularly pertinent in the context of amnestic MCI patients. Participants in our study could be arranged along a continuum of gradual decline regarding switching in the SVF task, with NC individuals performing better than aMCI and AD patients, and aMCI patients surpassing those with dementia (NC > aMCI > AD).

Those diagnosed with aMCI had fewer switches and clusters in both VF tasks compared to NC participants. This is in accordance with the findings of Mueller et al. [44], as they observed that the aMCI group performed worse on total word production and switching in comparison to NC, a finding that was more pronounced in the aMCI subgroup that experienced deficits in both executive function and memory. Primarily, their performance closely resembled that of individuals with dementia, a finding contrary to other investigators proposing performance akin to that of healthy older adults [14,27]. On the other hand, a longitudinal study observed a reduction in switches among healthy individuals who later developed AD after a span of 5 years [24]. Others have also found aMCI (especially multi-domain) impairments in both total word count, clustering and switching used in VF, although the available literature on this is limited [27,28,44]. These conflicting findings could be accounted for by a number of factors, including the operational definition of aMCI (including criteria and tasks employed), differences in participant demographics, and the extent of cognitive decline within each study’s participants. Moreover, it is worth mentioning that our sample was larger compared to that of other studies, and this might explain the differences in our results.

Amnestic MCI patients outperformed patients with dementia solely in SVF switches, while naMCI had better performance than AD in both VF switches and SVF clusters. These findings indicate that individuals with aMCI present language and executive decline similar to those seen in dementia, possibly due to deficits in both temporal and frontal lobe functions. The findings of prior studies, that the phonemic task remains relatively unaffected in the early stages [27,45], did not align with our results, since aMCI performed worse than NC on PVF switches and clusters. Nevertheless, there are studies that presented comparable levels of phonemic and semantic deficits in individuals with aMCI [14,44,46] suggesting that VF is a task that places substantial demands on both semantic storage and retrieval abilities, and these abilities become compromised in a way that MCI patients can no longer effectively compensate for. Our findings are also in accordance with the findings of Mueller et al. [44], as they observed that the low performance of the aMCI group on total PVF and SVF word production and PVF switching was particularly pronounced within the subgroup of aMCI individuals exhibiting deficits in both executive function and memory. Interestingly, they found a strong correlation between the total word production and switching ability in PVF, suggesting that these two measures may be reliant on the same cognitive processes. It is possible that the poorer performance of the aMCI group was driven by the aMCI multi-domain subgroup, as they performed worse than those with single-domain naMCI in both VF switches. In our aMCI subgroup, there was a greater prevalence of multi-domain patients in comparison to those with single-domain aMCI. In contrast, the two subgroups within the naMCI group showed a more comparable distribution. Previous researchers have suggested that aMCI multi-domain patients display a performance profile that is more akin to individuals with AD and less similar to those with single-domain MCI [14,27,47]. Additionally, this subgroup has been identified as having a heightened susceptibility to the development of AD dementia [48,49,50]. Furthermore, with the exception of the number of clusters in PVF, NC individuals demonstrated superior performance compared to those with dementia across all indices. While a significant difference existed between aMCI and NC participants, this could not be replicated between NC and AD patients, rendering it devoid of its clinical relevance.

Moreover, naMCI patients performed similarly to healthy individuals in all VF strategies. Previous studies, albeit limited in number, have suggested that naMCI patients perform worse compared to healthy individuals in total SVF and PVF word production [14] and PVF switches [27]. Conversely, some studies have found that naMCI patients exhibited a performance similar to that observed in NC individuals [13]. Switching deficits have also been observed in these patients [27], in contrast to the present findings. Moreover, in our study, naMCI individuals exhibited comparable VF strategies performance to those with aMCI, and there was only a slight tendency noted in SVF cluster number, favoring the naMCI group. The differences observed between our findings and those of other studies may potentially be attributed to the variations in sample sizes. For instance, Weakley et al. [27] included 74 aMCI and 16 naMCI participants. Similarly, the majority of other studies encompassing both subgroups included about 70–80 MCI patients [14,25], and one study even included 210 MCI individuals [13]. In contrast, the present study included a larger sample of 238 MCI individuals (146 aMCI and 92 naMCI), and we included a substantial number and proportion of both single- and multi-domain naMCI patients: 41 and 51, respectively; the latter sets our study apart from most other studies that did not differentiate the naMCI subgroups. The observed differences might alternatively be explained by discrepancies in demographic characteristics and the extent of impairment across the studies.

Additionally, a comparison was conducted between single- and multi-domain aMCI and naMCI patients on VF strategies. The analysis revealed noteworthy differences in the number of switches in both tasks, favoring single-domain naMCI patients over multi-domain aMCI patients. Similarly, single-domain naMCI patients produced more switches on SVF compared to multi-domain naMCI. These findings align with prior research [14,27,47], which consistently highlighted the decline in VF observed in multi-domain MCI patients. This decline is believed to stem from compromised executive functioning and more extensive brain pathology. These results were expected given the importance of executive functioning in VF tasks, encompassing aspects such as retrieval, initiation, inhibition, and particularly switching (shifting ability) [51]. It can be suggested that multiple-domain MCI presents with more widespread, or a higher degree of brain dysfunction compared to those with single-domain. This association has been established in studies that have indicated an elevated likelihood of these patients transitioning to AD dementia [49,50]. Finally, no significant group differences were observed with respect to errors. Other investigators have also failed to detect any differences in errors between controls and those who later progressed to AD [24].

Nevertheless, it is important to highlight several constraints inherent in our study. To begin with, the identification of individuals with MCI and dementia relied solely on clinical criteria, devoid of any reliance on biomarkers or imaging modalities. This absence of objective markers introduces an inherent susceptibility to misclassification, thereby compelling us to deduce that Verbal Fluency (VF) strategies should not be employed in isolation for diagnostic purposes. In light of this limitation, the possibility of misclassification bias in our study remains.

Also, dementia diagnoses other than AD were not examined in the present analyses due to the small number of diagnosed participants in those categories. Finally, the relatively short follow-up period of approximately 3 years might have underpowered several analyses. Overall, our study addressed a significant gap in VF literature by examining performance differences in VF strategies across the continuum of neurocognitive decline, including various subtypes of MCI. Additionally, we leveraged a large sample size randomly selected from both provincial and metropolitan areas in Greece as part of the HELIAD study. This extensive dataset enables us to draw broader conclusions applicable to the entire elderly Greek population [7]. Our study underscores the importance of assessing strategies in addition to the conventional scoring method involving total words on the VF task, namely clustering, switching, and errors. We observed a continuum of progressive SVF switching deterioration in our participants (NC > aMCI > AD). Future longitudinal assessments of the VF strategies within the continuum of neurocognitive decline are necessary, including individuals with subjective cognitive decline. Additionally, it is important that our findings pertaining to the VF strategies’ ability to differentiate among older adults with different cognitive statuses could be validated in independent cohorts in future investigations.

## 5. Conclusions

Verbal fluency strategies play an important role in distinguishing between healthy older adults and those with amnestic MCI, as well as between single- and multi-domain MCI cases. Our findings indicate a gradual decline in switching ability within the SVF task, with NC individuals surpassing both aMCI and AD patients, and aMCI patients performing better than those with dementia. The observed difficulties in SVF switching suggest compromised executive function and may serve as an indicator for diagnosing multi-domain MCI, particularly the multi-domain subtype of aMCI.

## Figures and Tables

**Table 1:** Baseline characteristics of NC, aMCI, naMCI, and AD individuals.

	Parameter	NC (n = 1607)	aMCI (n = 146)	naMCI (n = 92)	AD (n = 79)
	Age in years (N = 1924)	73.31 ± 5.19	74.84 ± 5.20	75.91 ± 5.26	79.96 ± 6.36
	Gender (M/F) (N = 1924)	643/964 (40.0%/60.0%)	65/81 (45.5%/55.5%)	39/53 (42.4%/57.6%)	32/47 (40.5%/59.5%)
	Years of education (N = 1923)	8.17 ± 4.86	7.20 ± 5.44	6.92 ± 4.78	6.87 ± 5.30
Semantic task (N = 1873)	Production	15.70 ± 4.98	11.30 ± 5.12	13.58 ± 5.48	7.77 ± 4.73
	Number of clusters	2.27 ± 1.31	1.62 ± 1.16	2.07 ± 1.23	1.13 ± 1.06
	Number of switches	9.70 ± 4.71	7.28 ± 4.23	8.07 ± 4.55	4.29 ± 3.00
	Number of errors	0.62 ± 1.51	0.65 ± 2.15	0.49 ± 1.34	0.30 ±0.81
	Number of perseverations	0.57 ± 1.01	0.60 ± 0.99	0.48 ± 0.87	0.29 ± 0.66
Phonemic task (N = 1815)	Production	7.39 ± 4.52	4.92 ± 4.14	5.72 ± 3.67	3.57 ± 3.69
	Number of clusters	0.33 ± 0.74	0.08 ± 0.27	0.16 ± 0.45	0.13 ± 0.34
	Number of switches	6.53 ± 4.10	4.68 ± 4.06	5.31 ± 3.12	3.28 ± 3.53
	Number of errors	0.26 ± 0.75	0.30 ± 0.76	0.27 ± 0.56	0.53 ± 1.26
	Number of perseverations	0.36 ± 0.83	0.22 ± 0.56	0.16 ± 0.42	0.18 ± 0.79

NC: normal cognition; aMCI: amnestic mild cognitive impairment; naMCI: non-amnestic MCI; AD: Alzheimer’s disease; N: number of participants with available data per variable; M/F: male/female.

**Table 2:** Differences in verbal fluency strategies (with 95% confidence intervals and corresponding *p*-values) among (and between) NC, aMCI, naMCI, and AD individuals. A total of 1814 participants with available data for all dependent and independent variables (1519 NC, 138 aMCI, 90 naMCI, 67 AD) underwent analysis using multivariate general linear models (GLMs), adjusted for the mean age and formal education of our sample, as well as the average sex effect.

	Parameter	NC vs. aMCI	NC vs. naMCI	NC vs.AD	aMCI vs. naMCI	aMCI vs. AD	naMCI vs. AD
Semantic task	Number of clusters	**0.57 (0.27, 0.87), <0.001**	0.11 (−0.25, 0.48), 1.000	**0.90 (0.47, 1.33), <0.001**	−0.45 (−0.91, 0.00), 0.051	0.34 (−0.17, 0.84), 0.480	**0.79 (0.24, 1.33), >0.001**
	Number of switches	**1.71 (0.74, 2.69), <0.001**	0.83 (−0.36, 2.02), 0.389	**4.21 (2.81, 5.61), <0.001**	−0.88 (−2.36, 0.60), 0.694	**2.50 (0.85, 4.14), <0.001**	**3.38 (1.61, 5.14), <0.001**
	Number of errors	−0.04 (−0.41, 0.32), 1.000	0.18 (−0.41, 0.32), 1.000	0.42 (−0.11, 0.95), 0.208	0.22 (−0.33, 0.78), 1.000	0.46 (−0.15, 1.08), 0.284	0.24 (−0.42, 0.91), 1.000
	Number of perseverations	−0.04 (−0.27, 0.20), 1.000	0.09 (−0.20, 0.37), 1.000	0.26 (−0.09, 0.59), 0.282	0.12 (−0.24, 0.48), 1.000	0.29 (−0.10, 0.69), 0.310	0.17 (−0.26, 0.60), 1.000
Phonemic task	Number of clusters	**0.22 (0.06, 0.37), 0.002**	0.13 (−0.07, 0.32), 0.515	0.15 (−0.08, 0.38), 0.504	−0.09 (−0.33, 0.15), 1.000	−0.07 (−0.33, 0.20), 1.000	0.02 (−0.27, 0.31), 1.000
	Number of switches	**1.37 (0.56, 2.19), <0.001**	0.50 (−0.50, 1.49), 1.000	**2.33 (1.16, 3.50), <0.001**	−0.88 (−2.11, 0.36), 0.363	0.96 (−0.42, 2.33), 0.398	**1.83 (0.36, 3.31), 0.006**
	Number of errors	−0.04 (−0.22, 0.14), 1.000	−0.01 (−0.23, 0.22), 1.000	−0.26 (−0.52, 0.00), 0.055	0.04 (−0.14, 0.22), 1.000	−0.22 (−0.52, 0.08), 0.355	−0.25 (−0.58, 0.08), 0.258
	Number of perseverations	0.12 (−0.06, 0.30), 0.494	0.17 (−0.06, 0.39), 0.293	0.14 (−0.13, 0.40), 1.000	0.05 (−0.23, 0.33), 1.000	0.02 (−0.29, 0.33), 1.000	−0.03 (−0.36, 0.30), 1.000

NC: normal cognition; aMCI: amnestic mild cognitive impairment; naMCI: non-amnestic MCI; AD: Alzheimer’s disease; positive mean differences conventionally represent a superior performance of first group of participants as quoted at the top of each column, while negative mean differences correspond to a superior performance of the second group of participants as quoted at the top of each column; bold denotes statistical significance of the results.

**Table 3:** Differences in verbal fluency strategies (with 95% confidence intervals and corresponding *p*-values) among (and between) individuals with single-domain aMCI, multi-domain aMCI, single-domain naMCI and multi-domain naMCI, adjusted to the mean age and formal education of our sample as well as to the average sex effect.

	Parameter	maMCI vs. aMCI	maMCI vs. naMCI	maMCI vs. mnaMCI	aMCI vs. naMCI	aMCI vs. mnaMCI	mnaMCI vs. naMCI
Semantic task	Number of clusters	−0.17 (−0.76, 0.42), 1.000	−0.52 (−1.15, 0.07), 0.117	−0.52 (−1.07, 0.04), 0.080	−0.35 (−1.05, 0.34), 1.000	−0.35 (−1.00, 0.31), 0.955	−0.01 (−0.67, 0.66), 1.000
	Number of switches	−1.87 (−3.77, 0.03), 0.057	**−2.85 (−4.77, −0.93), 0.001**	−0.32 (−2.11, 1.47), 1.000	−0.98 (−3.22, 1.26), 1.000	1.55 (−0.56, 3.67), 0.323	**−2.53 (−4.68, 0.38), 0.012**
	Number of errors	0.70 (−0.24, 1.63), 0.292	0.53 (−0.41, 1.47), 0.806	0.32 (−0.56, 1.19), 1.000	−0.16 (−1.26, 0.93), 1.000	−0.38 (−1.42, 0.66), 1.000	0.21 (−0.84, 1.27), 1.000
	Number of perseverations	−0.19 (−0.66, 0.28), 1.000	0.20 (−0.27, 0.68), 1.000	0.02 (−0.42, 0.46), 1.000	0.39 (−0.17, 0.94), 0.379	0.20 (−0.32, 0.73), 1.000	0.18 (−0.35, 0.71), 1.000
Phonemic task	Number of clusters	−0.07 (−0.25, 0.10), 1.000	−0.15 (−0.33, 0.03), 0.116	−0.07 (−0.24, 0.09), 1.000	−0.08 (−0.28, 0.12), 1.000	−0.00 (−0.19, 0.19), 1.000	−0.08 (−0.27, 0.12), 1.000
	Number of switches	−0.85 (−2.42, 0.72), 0.899	**−1.65 (−3.23, −0.06), 0.037**	−0.78 (−2.26, 0.69), 0.942	−0.79 (−2.64, 1.06), 1.000	0.07 (−1.69, 1.82), 1.000	−0.86 (−2.63, 0.92), 1.000
	Number of errors	−0.09 (−0.43, 0.26), 1.000	0.01 (−0.34, 0.36), 1.000	−0.03 (−0.35, 0.30), 1.000	0.10 (−0.31, 0.50), 1.000	0.06 (−0.32, 0.45), 1.000	0.03 (−0.36, 0.42), 1.000
	Number of perseverations	−0.22 (−0.47, 0.03), 0.136	0.03 (−0.23, 0.21), 1.000	−0.02 (−0.26, 0.21), 1.000	0.24 (−0.05, 0.54), 0.183	0.41 (−0.09, 0.47), 0.408	0.05 (−0.23, 0.33), 1.000

aMCI: single-domain amnestic mild cognitive impairment; naMCI: single-domain non-amnestic MCI; maMCI: multi-domain aMCI; mnaMCI: multi-domain naMCI; positive mean differences conventionally represent a superior performance of first group of participants as quoted at the top of each column, while negative mean differences correspond to a superior performance of the second group of participants as quoted at the top of each column; bold denotes statistical significance of the results.

## Data Availability

The data that support the findings of this study are available from the corresponding author, upon reasonable request.
